# Influence of Decreased Temperature of Tensile Testing on the Annealing-Induced Hardening and Deformation-Induced Softening Effects in Ultrafine-Grained Al–0.4Zr Alloy

**DOI:** 10.3390/ma15238429

**Published:** 2022-11-26

**Authors:** Tatiana S. Orlova, Aydar M. Mavlyutov, Maxim Yu. Murashkin, Nariman A. Enikeev, Alexey D. Evstifeev, Dinislam I. Sadykov, Michael Yu. Gutkin

**Affiliations:** 1 Ioffe Institute, Russian Academy of Sciences, Polytekhnicheskaya Str. 26, 194021 St. Petersburg, Russia; 2 Laboratory of Dynamics and Extreme Characteristics of Promising Nanostructured Materials, Saint Petersburg State University, Universitetskiy Pr. 28, 198504 St. Petersburg, Russia; 3 Center for Design of Functional Materials, Ufa University of Science and Technology, Z. Validi Str. 32, 450076 Ufa, Russia; 4 Institute of Advanced Data Transfer Systems, ITMO University, Kronverkskiy Pr. 49, 197101 St. Petersburg, Russia; 5 Institute for Problems in Mechanical Engineering, Russian Academy of Sciences, Bolshoj Pr. 61, V.O., 199178 St. Petersburg, Russia; 6 High School of Mechanics and Control Processes, Peter the Great St. Petersburg Polytechnic University, Polytekhnicheskaya Str. 29, 195251 St. Petersburg, Russia

**Keywords:** ultrafine-grained aluminum-zirconium alloys, mechanical properties, annealing-induced hardening, deformation-induced softening, low temperatures

## Abstract

The influence of decreased temperature of tensile testing on annealing-induced hardening (AIH) and deformation-induced softening (DIS) effects has been studied in an ultrafine-grained (UFG) Al–Zr alloy produced by high-pressure torsion. We show that the UFG Al–Zr alloy demonstrates a DIS effect accompanied by a substantial increase in the elongation to failure *δ* (up to *δ* ≈ 30%) depending on the value of additional straining. Both the AIH and DIS effects weaken with a decrease in the tensile test temperature. The critical deformation temperatures were revealed at which the AIH and DIS effects are suppressed. The activation energy *Q* of plastic flow has been estimated for the UFG Al–Zr alloy in the as-processed, subsequently annealed and additionally strained states. It was shown that the annealing decreases the *Q*-value from ~80 kJ/mol to 23–28 kJ/mol, while the subsequent additional straining restores the initial *Q*-value. Alloying with Zr results in the expansion of the temperature range of the AIH effect manifestation to lower temperatures and results in the change in the *Q*-value in all of the studied states compared to the HPT-processed Al. The obtained *Q*-values and underlying flow mechanisms are discussed in correlation with specific microstructural features and in comparison to the UFG Al.

## 1. Introduction

Aluminum alloys are widely used in different industrial fields, including electrical engineering, due to a good combination of properties such as low weight, good electrical conductivity, and corrosion resistance. The main disadvantage of aluminum alloys is their relatively low mechanical strength. In recent years, severe plastic deformation (SPD) techniques have been extensively investigated [1,2]. These methods allow processing of bulk metallic samples with ultrafine-grained (UFG) structures, which provide increased mechanical strength due to grain boundary strengthening and strain hardening mechanisms [1,2].

Among the numerous aluminum alloys, the Al–Zr system is of special interest. The small addition of zirconium (up to 1 wt.%) promotes formation of a thermally stable structure in the system [3,4,5,6,7,8]. This is achieved by the precipitation of Al_3_Zr nanoparticles that serve as effective barriers preventing the migration of grain boundaries (GBs) upon heating [3].

Previously, we studied the influence of high-pressure torsion (HPT) on the microstructure and mechanical properties of the Al–0.4Zr alloy, as well as the heat resistance of the obtained mechanical characteristics of the alloy in the UFG state to short-term annealing (1 h) at temperatures up to 280 °C [9]. It was shown that the annealing of the UFG Al–0.4Zr alloy for 1 h at temperatures of 230–280 °C results in a unique combination of functional properties—the ultimate tensile strength *σ*_UTS_ = 200–250 MPa and the electrical conductivity equals 49–50% IACS [9]—which demonstrates the high potential of such an alloy for use under elevated temperatures, for example, as a wire material for high-voltage power lines. Moreover, the effect of significant additional strengthening after low-temperature annealing of the UFG Al–0.4Zr alloy was revealed [9]. The maximum increases in the yield stress (*σ*_0.2_ ≈ 65%) and the ultimate tensile strength (*σ*_UTS_ ≈ 30%) were reached as a result of annealing at 230 °C for 1 h. At the same time, the plasticity of the UFG alloy remained at a sufficiently high level of ~12%.

A similar annealing-induced hardening (AIH) effect was earlier revealed for the UFG commercially pure (CP) Al processed by HPT [10]. Annealing-induced hardening in UFG CP Al was accompanied by a drastic drop in plasticity to ~1%. This effect is not typical for coarse-grained (CG) structures and is associated with grain refinement under HPT processing, which leads to a high density of high-angle grain boundaries (HAGBs) in the UFG structure [10,11]. According to the theoretical model [11], the AIH and a decrease in plasticity of UFG CP Al are associated with relaxation of the HAGB structure, resulting in a decrease in the density of extrinsic grain boundary dislocations (EGBDs). This leads to a decrease in the number of EGBDs in their pile-ups at triple junctions which are formed under external loading and, as a result, leads to the hindering of dislocation emission from GBs for the implementation of plastic flow. Additional HPT deformation to 0.25 revolutions of the samples with a post-annealing relaxed structure led to an increase in the ductility of UFG Al to values typical for the CG state (~34%) while maintaining the high level of yield strength (~130 MPa) and ultimate tensile strength (~180 MPa) [10]. The effect of ductility enhancement due to additional deformation—deformation-induced softening (DIS)—was explained by a ~3-fold increase in the density of dislocations in GBs and near-boundary regions after such additional deformation [10,11].

For the first time, the effects of hardening by annealing and softening due to additional deformation after annealing in UFG aluminum were reported in ref. [12] for CP Al processed by accumulative roll bonding (ARB). Increase in the yield stress (*σ*_0.2_) by ~9% was demonstrated after annealing of ARB-processed Al at 150 °C for 0.5 h. The authors explained the increase in strength by a decrease in the sources of mobile lattice dislocations due to their sinkage during annealing into closely spaced HAGBs, and the increase in plasticity after additional deformation was related to introducing new dislocations into the grain interior (by an increase of the number of dislocation sources in grains) [12].

Recently it has been found [13] that the AIH effect in HPT-processed Al decreases with the decreasing temperature of deformation testing *T*_def_ and, moreover, completely disappears at *T*_def_ ~193 K and ~223 K for *σ*_0.2_ and *σ*_UTS_, respectively, whereas the suppression of ductility by annealing is kept up to 193 K at least. The study of the influence of test temperature on AIH and DIS effects is important from the point of view of the fundamental physics of mechanical behavior of UFG metals and alloys. Since decreasing the temperature affects the thermally-activated processes of plastic deformation, their comparative studies at low and room temperatures therefore allow for a deeper understanding of the physical nature of the AIH and DIS effects.

In this work, we demonstrate for the first time the effect of an increase in plasticity as a result of a small additional deformation (DIS effect) for the Al–0.4Zr alloy with the UFG structure relaxed by low-temperature annealing, as well as the effect of decreased deformation temperatures, including the cryogenic temperatures, on this DIS effect and the earlier observed effect of AIH in this alloy [14].

## 2. Materials and Experimental Procedures

The alloy with the following chemical composition: 0.39 Zr, 0.24 Fe, 0.02 Si, 0.02 Zn, and rest Al wt. % was studied (the Al–0.4Zr alloy). The Al–Zr alloy in the shape of rods 9.5 mm in diameter was obtained by combined casting and rolling [15,16]. The materials were supplied by UC RUSAL (Moscow, Russia). Cylinder-shaped billets were cut from the initial rods and subjected to HPT, with 10 revolutions under the hydrostatic pressure of 6 GPa at room temperature (RT). As a result, disc-shaped samples with a diameter of 20 mm and a thickness of 1.2 mm were obtained (the samples are denoted as HPT state). The true strain at the distance of 5 mm from the disc center was *ε_tr_* ≈ 6.6 [2]. After HPT-processing, some samples were subjected to two types of treatments: (1) annealing at 503 K for 1 h (HPT + AN state) and (2) annealing at 503 K for 1 h and additional HPT deformation at the same pressure and temperature to 0.25 (HPT + AN + 0.25 HPT state), 0.5, and 0.75 of revolutions. The annealing temperature 503 K was chosen because it corresponds to the peak AIH effect in the Al–Zr alloy [14].

For uniaxial tension tests, blade-shaped samples with a working region of (6 × 2) mm^2^ were cut from the obtained discs using an electrical discharge machine [10]. Uniaxial tension was performed using a Shimadzu AG-XD Plus test machine at various temperatures *T*_def_ in the range from 77 (in liquid nitrogen) to 293 K in a chamber cooled by liquid nitrogen vapor. The values of *T*_def_ were fixed with accuracy of ±0.5 K. The strain rate was 5 × 10^−4^ s^−1^. At least three samples were tested for each state and each test temperature. Using the measured stress–strain diagrams, the conventional yield stress (*σ*_0.2_) (the stress corresponding to a relative elongation of 0.2%), the ultimate tensile strength (*σ*_UTS_), the elongation to failure (*δ*), and the uniform elongation (*δ*_1_) were determined. A strain rate jump experiment was performed with the strain rate change from 5 × 10^−4^ s^−1^ to 1 × 10^−3^ s^−1^ and vice versa.

The X-ray diffraction (XRD) measurements were performed on the Bruker D2 PHASER diffractometer using *θ*–2*θ* Bragg–Brentano geometry and CuKα irradiation with a scan increment of 0.02°. Instrumental broadening was accounted for by measuring an Al_2_O_3_ standard sample. The obtained X-ray profiles were analyzed in terms of the Rietveld refinement technique realized in MAUD software [17] to calculate lattice parameter, coherent domain size, and microstrain values. The dislocation density was deduced from the measured values as proposed in [18].

The electron backscatter diffraction (EBSD) studies were performed on the scanning electron microscope Zeiss Merlin with a scanning step of 0.2 μm. For each diffraction pattern, seven Kikuchi bands were used for indexing. The EBSD maps, which included at least 1500 grains for each state, were used to determine the average grain size (*d*_av_), fraction of HAGBs (*f*_HAGB_), and the average grain boundaries misorientation angle (*θ*_av_). The area of each grain was approximated by the circle area, the diameter of which was taken as a grain size [19].

The microstructure was studied using a Jeol JEM-2100 (JEOL Ltd, Tokyo, Japan) transmission electron microscope (TEM) operated at 200 kV. Thin foils for the TEM observations were prepared by mechanical polishing with subsequent double-jet electropolishing following the regime used in [14].

## 3. Results and Discussion

### 3.1. Microstructure Characterization

**Figure 1:**
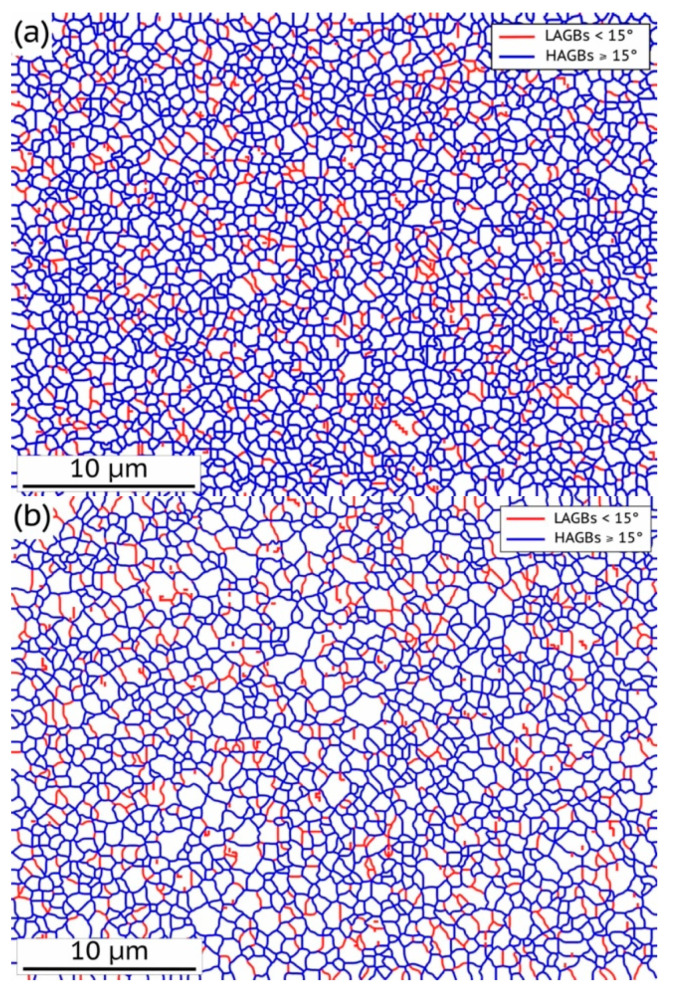
HAGBs (blue lines) and LAGBs (red lines) manifestation in the microstructure of the Al–0.4Zr alloy subjected to HPT (**a**) and to subsequent annealing (**b**).

The microstructure of the samples of as-received, HPT and HPT + AN states was studied by electron backscatter diffraction, and transmission electron microscopy in our previous works [9,14], the results of EBSD studies are presented in Table 1. The initial state of the Al–0.4Zr alloy was characterized by subgrains elongated in the rolling direction with an average length of ~1800 and a width of ~1000 nm (the HAGB fraction was 25%) [14]. The EBSD data for HPT and HPT + AN states have been additionally treated and the additional information on the grain boundary misorientation is presented in Figure 1.

The samples in the HPT state had a homogeneous UFG structure consisting of equiaxed grains with an average size of ~835 nm, and an HAGB fraction of 82%. The average grain size only slightly increased (Table 1) and the fraction of high-angle grain boundaries practically did not change after annealing (Figure 1, Table 1), which indirectly indicates the efficiency of the pinning of GBs and their triple junctions by impurity segregations and/or nanoclusters/nanoprecipitates. The formation of nanosized (10–15 nm) Al_3_Zr precipitates on some GBs was observed in in situ annealing experiments in a scanning transmission electron microscope [20]. As shown earlier, in the Al–0.4Zr alloy in the initial state (before HPT processing), all Zr atoms were dissolved in aluminum matrix [4,9,14]. Annealing at 503 K led to the formation of a small amount of nanosized precipitates of the Al_3_Zr phase inside the grains [9,14].

X-ray diffraction analysis (XRD) of all of the investigated states was performed in the same measurement geometry on the same equipment for comparison in the present study. The microstructural parameters such as the lattice parameter *a*, the average size of coherent scattering domains *D*_XRD_, the level of microdistortions ⟨ε^2^⟩^1/2^, and the dislocation density *L*_dis_ were determined by X-ray diffraction analysis (Table 1). The dislocation density *L*_dis_ is 5.2 × 10^12^ m^−2^ in the HPT state. Subsequent annealing at 503 K caused a decrease of *L*_dis_ by approximately a factor of 3.7 (Table 1). Additional deformation after annealing to 0.25 and 0.75 revolutions led to a substantial increase in dislocation density (Table 1).

**Figure 2:**
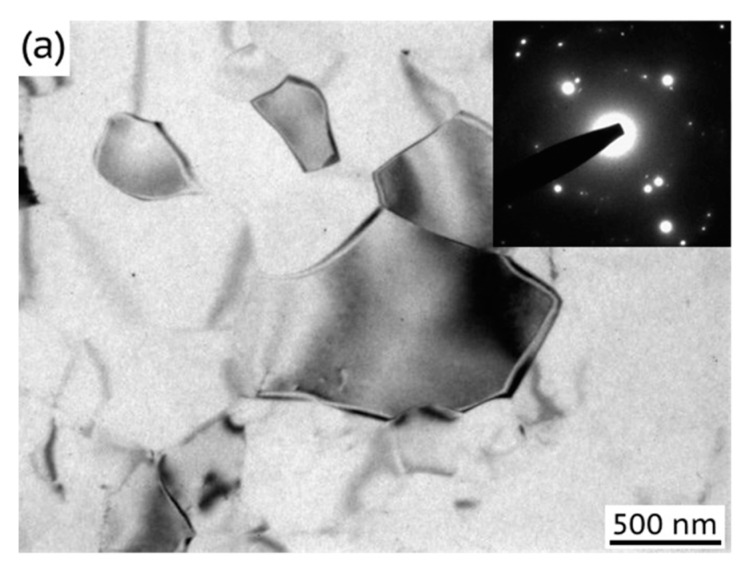

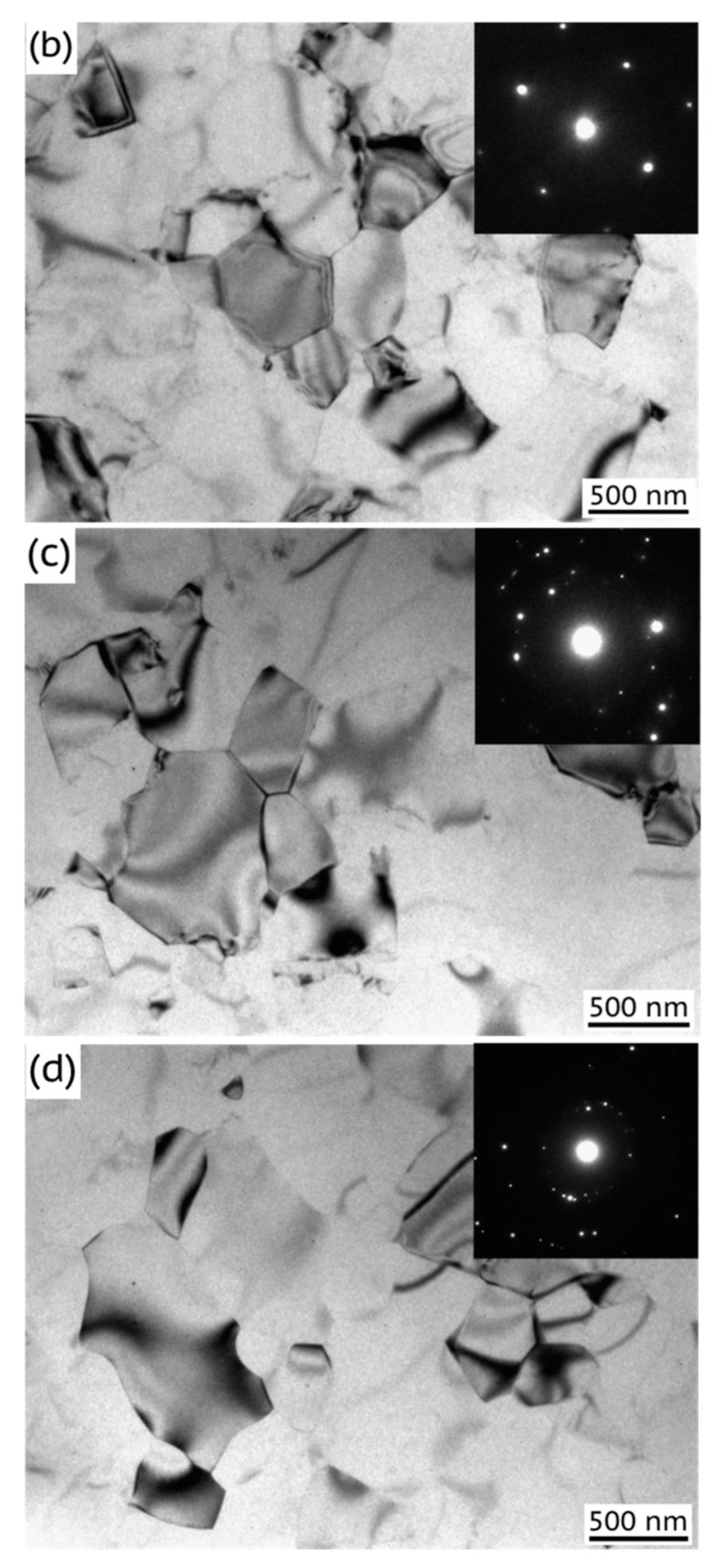
The microstructure of the Al–0.4Zr alloy subjected to HPT (**a**), subsequent annealing (**b**), and annealing and additional deformation by HPT to 0.25 (**c**) and 0.75 revolutions (**d**).

In addition, we performed a TEM study of the microstructure of the Al–0.4Zr alloy in HPT, HPT + AN, HPT + AN + 0.25 HPT, and HPT + AN + 0.75HPT states (Figure 2) and found that in all the states, the morphology of the grains is similar, and the grain interiors are nearly free of dislocation, except of few larger grains where dislocation walls/rare dislocations were seldom observed, which is not typical for the whole structure. This most probably means that the dislocation density determined by X-ray diffraction analysis refers mainly to GBs and near-boundary regions in all of the studied states and is consistent with the TEM observations in HPT and HPT + AN states in ref. [14]. Statistical observations of a good number of grains (~100 grains) in the TEM images did not reveal noticeable difference in grain morphology/size between the HPT + AN + 0.25 HPT and HPT + AN + 0.75 HPT states with different extents of additional deformation.

### 3.2. Mechanical Properties

**Figure 3:**
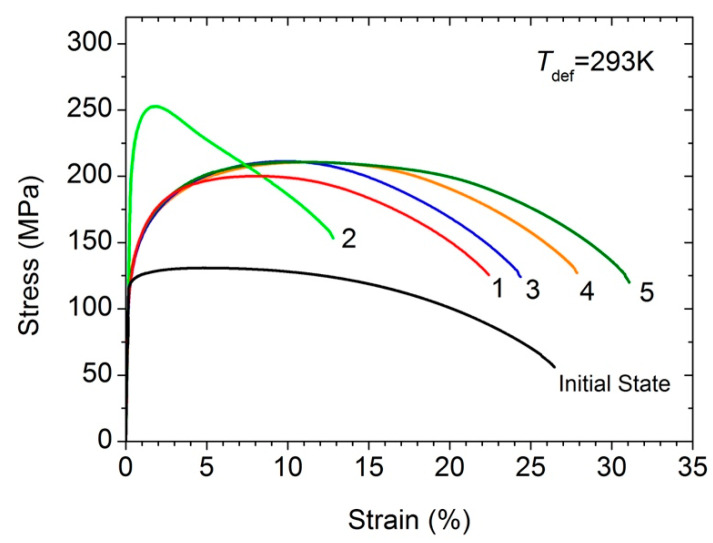
The stress–strain curves for the Al–0.4Zr alloy in the initial state, after HPT-processing (curve 1), after HPT-processing and subsequent annealing at 503 K for 1 h (curve 2), after HPT-processing, subsequent annealing, and additional HPT deformation to 0.25 (curve 3), 0.5 (curve 4), and 0.75 revolutions (curve 5).

Figure 3 shows the stress–strain diagrams obtained at room temperature for the samples of the Al–0.4Zr alloy in the initial state, after HPT treatment, subsequent annealing at 503 K for 1 h, and after annealing and additional HPT deformation to different values of 0.25, 0.5, and 0.75 revolutions. The alloy in the initial state has the yield stress of ~122 MPa, the ultimate tensile strength of ~131 MPa, and the high elongation to failure of ~26%, however the uniform elongation is only ~4%. As is seen, HPT processing leads to an increase in the yield stress to ~130 MPa, in the ultimate tensile strength to ~202 MPa, while the uniform elongation exceeds 8%. Subsequent annealing at 503 K for 1 h leads to an additional increase in the ultimate tensile strength and the yield stress to ~252 and ~223 MPa, respectively, but significantly reduces both the uniform elongation to ~1.5% and the total elongation to failure to ~13%, i.e., the AIH effect is observed. The hardening characteristics obtained after annealing of the HPT-processed Al-0.4 Zr alloy completely correspond to the results we obtained earlier [14].

Additional HPT to 0.25 revolutions leads to a decrease in the ultimate tensile strength and yield stress to ~213 and ~131 MPa, respectively, as well as to an increase in the elongation to failure and the uniform elongation to the values typical for the HPT state (*δ* ≈ 24% and *δ*_1_ ≈ 9.4%, see Table 2). This points to the restoration (after additional deformation) of the microstructure parameters that provide ductility similar to that in the HPT state. Such a feature is the dislocation density (Table 1), as in the case of CP Al [10,11].

Through increasing additional deformation to 0.75 revolutions, a tendency is observed for a further increase in ductility to *δ_1_* ≈ 10% and *δ* ≈ 30%, which exceed the plasticity of the alloy in the initial state. At the same time, the strength level is maintained (Figure 3) and this may be explained by introducing a larger number of dislocations (Table 1). Thus, despite the fact that the UFG Al–0.4Zr alloy after annealing (HPT + AN state) still possesses considerable ductility (*δ* ≈ 13%), the ductility significantly increases (to >30%) after subsequent additional deformation. This means that the UFG Al–0.4Zr alloy also exhibits the effect of DIS (yield stress reduction and increase in ductility) caused by deformation, which is not typical for coarse-grained metals and alloys.

**Figure 4:**
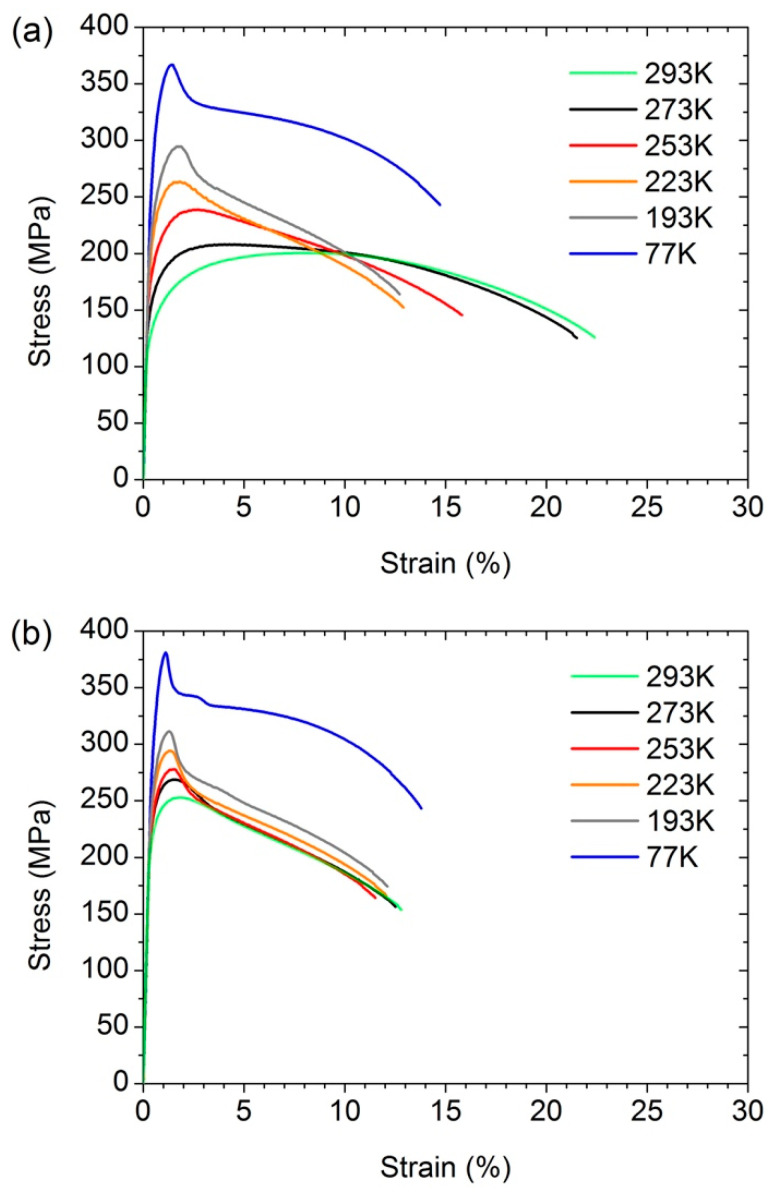

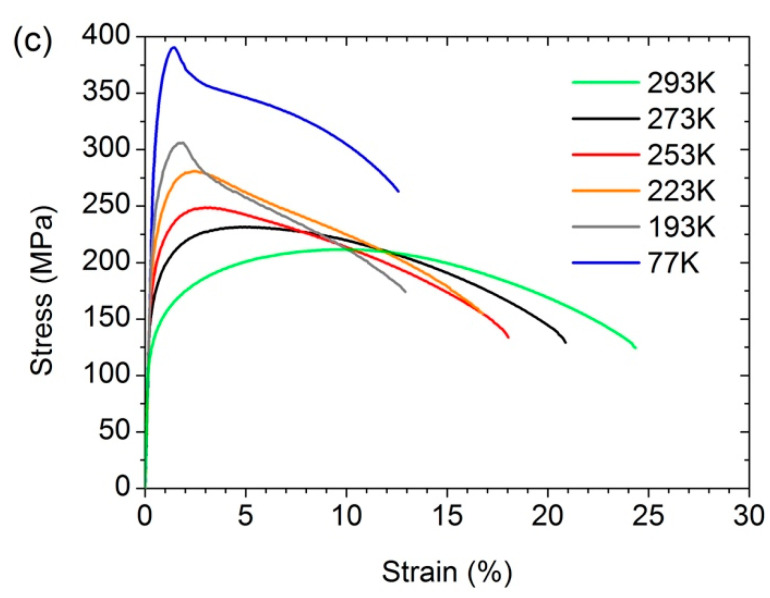
The stress–strain curves obtained at different deformation temperatures for the Al–0.4Zr alloy after (**a**) HPT-processing, (**b**) after HPT-processing and subsequent annealing at 503 K for 1 h, and (**c**) after HPT-processing, subsequent annealing, and additional HPT deformation to 0.25 revolutions.

**Figure 5:**
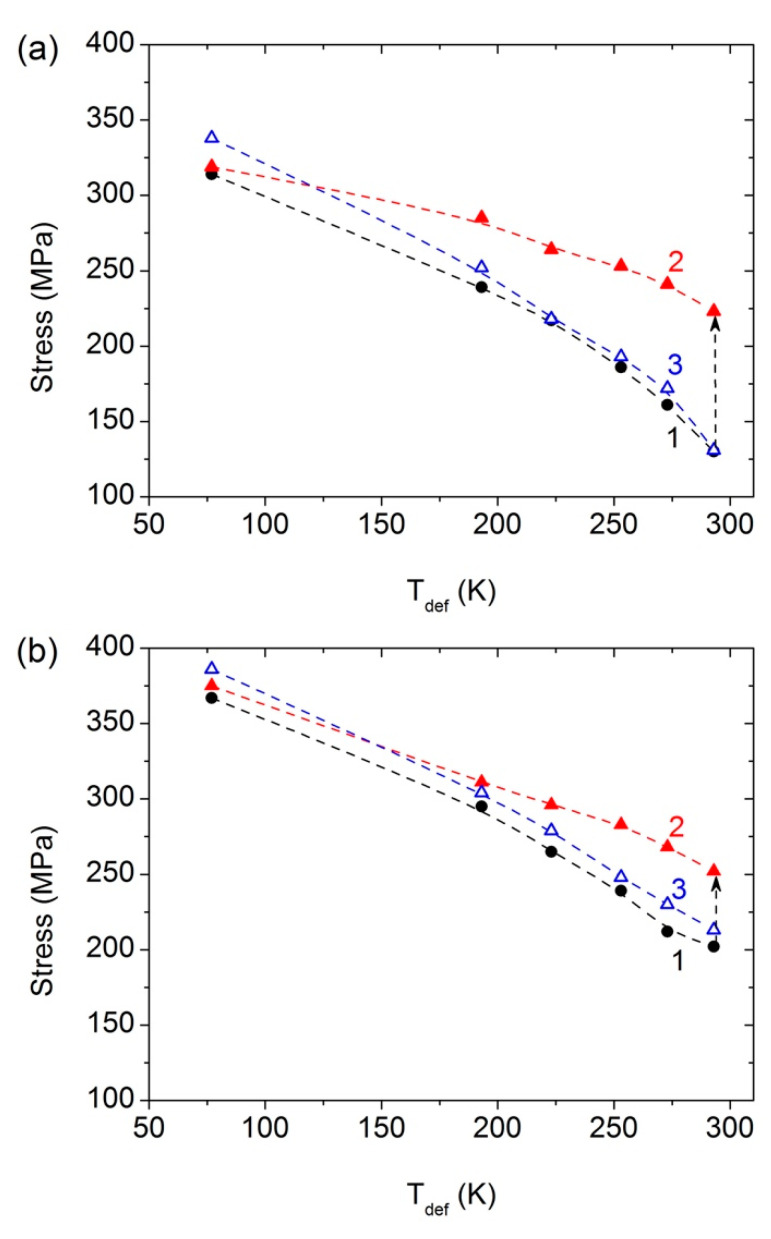

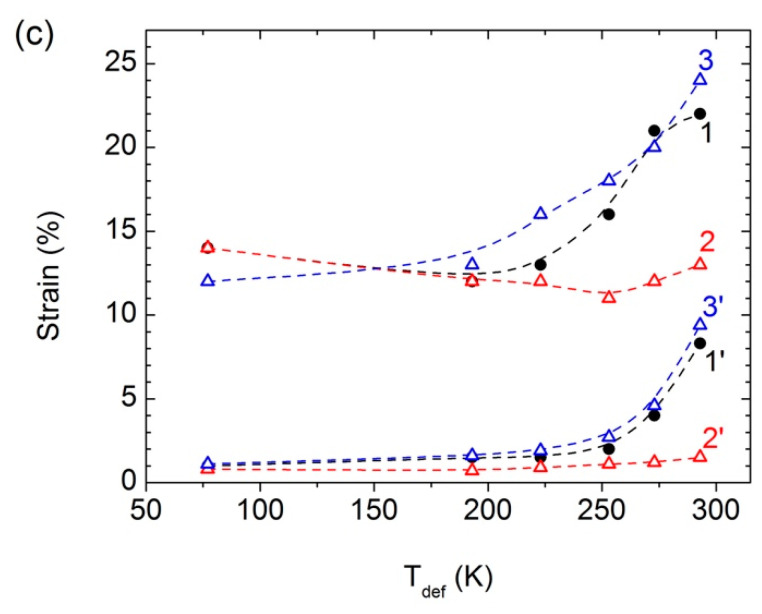
Dependence of mechanical properties—(**a**) yield stress, (**b**) ultimate tensile strength, and (**c**) total (curves 1–3) and uniform (curves 1′–3′) ductility—on the deformation temperature for the Al–0.4Zr alloy after HPT-processing (curves 1, 1′), after HPT-processing and subsequent annealing at 503 K for 1 h (curves 2, 2′), and after HPT-processing, annealing and additional HPT deformation to 0.25 revolutions (curves 3, 3′).

Furthermore, the influence of the temperature of tensile tests on the effects of AIH and DIS was investigated. Figure 4 shows the stress–strain curves obtained for the UFG Al–0.4Zr alloy in the HPT, HPT + AN, and HPT + AN + 0.25 HPT states at different deformation temperatures (*T*_def_). Figure 5 shows the dependences of the yield stress, ultimate tensile strength, uniform elongation, and total elongation to failure as a function of the deformation temperature for all three states of the alloy.

**Figure 6:**
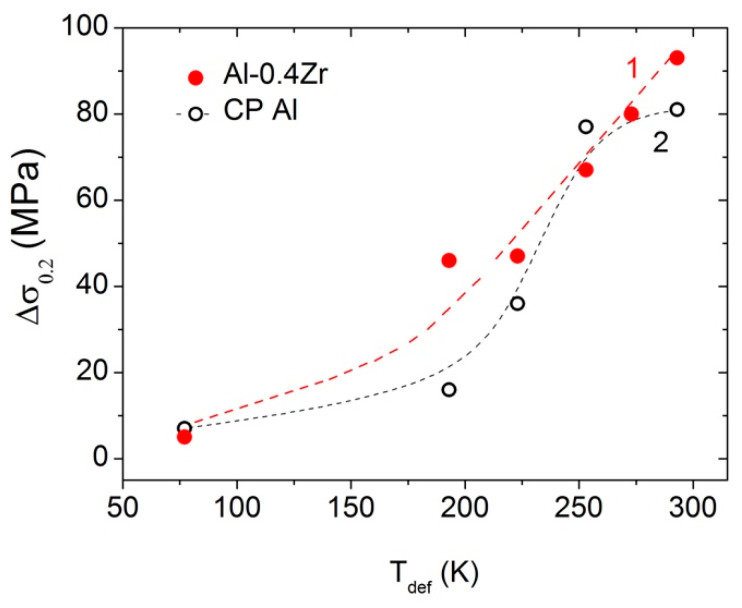
The annealing-induced yield stress increase (Δ*σ*_0.2_) versus the deformation temperature for the HPT-processed Al–0.4Zr alloy (curve 1) and HPT-processed CP Al (curve 2 plotted on the data of [13]).

The ultimate tensile strength and the yield stress increase, while the uniform deformation and the total ductility decrease with decreasing *T*_def_ in all of the the states under study (Figure 5, Table 2). In the UFG Al–0.4Zr alloy, the AIH effect recedes with decreasing temperature slower than in the UFG CP Al [13] (Figure 6). In the UFG CP Al, the AIH effect already disappears at *T*_def_ = 193 K, while in the UFG Al–0.4Zr alloy, it is still significant and gives Δσ_0.2_ ≈ 46 MPa (20%) at this deformation temperature. Since the main microstructural changes during annealing are a decrease in the dislocation density in GBs and nearby regions ([9,14], Table 1) and the formation of Al_3_Zr nanoprecipitates in some GBs [20], one can conclude that the presence of alloying Zr atoms and Al_3_Zr precipitates in GBs in the alloy leads to the expansion of the AIH effect not only towards higher temperatures up to 553 K [14], but also towards low temperatures, at least up to 193 K (Figure 5).

Similar to the AIH effect, the DIS effect also decreases with decreasing *T*_def_ (Figure 5c). Moreover, the DIS effect completely disappears at *T*_def_ = 193 K. These data correlate well with similar results previously obtained for CP Al [13] which may indicate a similar nature of this effect in CP Al and the Al–0.4Zr alloy.

It is noteworthy that in the HPT + AN state, both the values of *δ* and of *δ*_1_ are practically independent of *T*_def_ in the temperature range of 193–293 K (the range, where the AIH and DIS effects are preserved) and remain at the level of ≤1%, i.e., the samples show almost brittle behavior. Similar behavior was also observed for HPT-processed CP Al after annealing at *T*_AN_ = 423 K, corresponding to the peak AIH effect in this material [13].

### 3.3. Strain Rate Sensitivity and Activation Energy

The plastic deformation of metals and alloys can be described by the conventional power law [21,22]:

(1) $$\dot{\varepsilon }=A\sigma^{n}\exp(–Q/RT),$$

where *A* is a material constant, *n =* 1/*m* is the stress exponent, *m* is the strain rate sensitivity, *Q* is the activation energy, *R* is the gas constant and *T* is the absolute temperature. From Equation (1), *Q* can be expressed as [22]:

(2) $$Q=\frac{R}{m}\frac{\partial \ln\sigma }{\partial \left(1/T\right)}{|}_{\dot{\varepsilon }}$$

The derivative *∂*ln*σ/∂*(1*/T*) can be obtained from the ln*σ* versus 1*/T* curve at a certain strain rate $\dot{\varepsilon }$.

**Figure 7:**
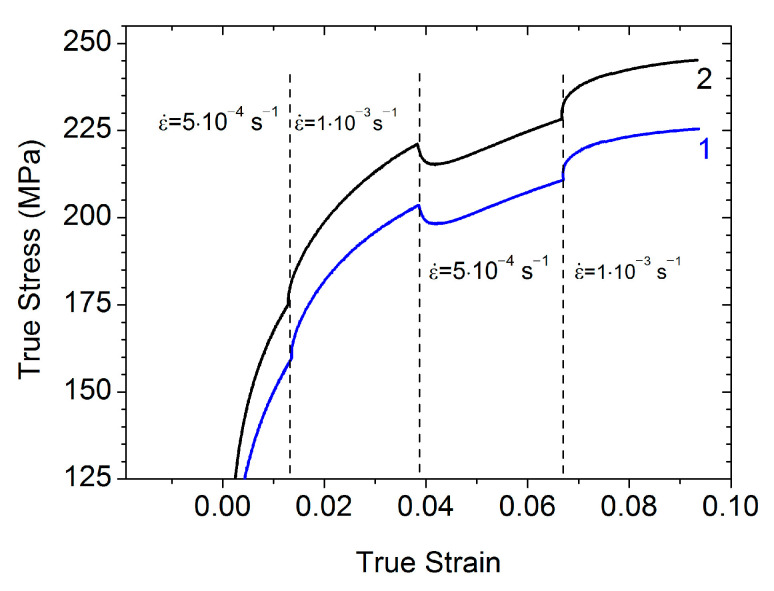
Strain-rate jump test at the base strain rate of $\;\dot{\varepsilon }=$ 5 × 10^−4^ s^−1^ for Al–0.4Zr alloy samples in HPT (curve 1) and HPT + AN + 0.25 HPT (curve 2) states.

Following [23], the strain-rate sensitivity coefficient *m* can be found from the strain-rate jump tests that for this study were performed at the base strain rate of $\;\dot{\varepsilon }=$ 5 × 10^−4^ s^−1^ with a jump to $\;\dot{\varepsilon }=$1 × 10^−3^ s^−1^ (Figure 7). The *m*-value determined from the stress-jump magnitude *m* = Δlnσ/Δln$\dot{\varepsilon }$ is equal to about 0.045 in both HPT and HPT + AN + 0.25 HPT states. The *m*-value obtained is comparable (slightly higher) with the *m*-values reported for HPT-processed Al (*m*~0.03) [24], ECAP-processed Al (*m*~0.04) [25,26], and the HPT-processed Al–Cu–Zr alloy (*m*~0.033–0.038) [27].

**Figure 8:**
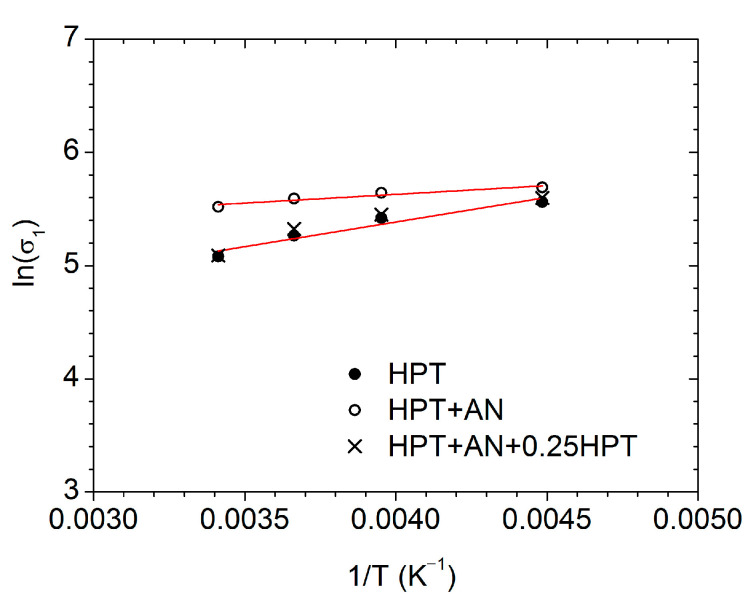
The natural logarithm of the flow stress at *ε* = 1% as a function of the inverse temperature for the Al–0.4Zr alloy in three different states.

Figure 8 displays the variations of ln *σ*_1_ with 1/*T* in the temperature range of the manifestation of the AIH and DIS effects at the true strain of 1% and $\dot{\varepsilon }$ = 5 × 10^−4^ s^−1^ for all of the studied states: HPT, HPT + AN and HPT + AN + 0.25. The obtained values of *Q* are shown in Table 3. The *Q*-value is equal to ~80 kJ/mol for both the HPT and HPT + AN + 0.25 HPT states, whereas in the HPT + AN state, the *Q*-value (~23 kJ/mol) is much lower. For comparison, similarly to ref. [24], we also estimated the *Q*-value from analysis of *σ*_0.2_ (*T*) behavior (for deformation *ε* = 0.2%). The obtained *Q*-values in both estimations (for *ε* = 0.2% and 1%) are nearly the same (Table 3).

We also analyzed the stress–strain curves obtained earlier for HPT-processed CP Al [14] in a similar way and obtained the *Q*-value of 95 kJ/mol for both HPT and HPT + AN + 0.25 HPT states (Table 3). The obtained *Q*-value for HPT-processed Al is in rather good agreement with the values extracted earlier from compression testing (*m* ≈ 0.03 and *Q* ≈ 82 kJ/mol) [25] and from depth-sensing micro- and nanoindentation (*m* ≈ 0.03 and *Q* ≈ 87 kJ/mol [24]). The slightly higher *Q* value for HPT-processed Al in our case may be due to the difference in the applied strain rate: 5 × 10^−4^ herein vs. 5 × 10^−3^ s^−1^ in ref. [24]. The *Q*- value can decrease with an increase in the strain rate at the same deformation temperature as was reported, for example, for an extruded Mg-5Zn-2.5Y-1Ce-0.5Mn alloy [22]. The temperature range is also different: 193–293 K in the present paper and 293–350 K in [24].

The obtained estimates of the activation energy of the plastic flow in the states before annealing, after annealing, and after annealing and additional deformation for the HPT-processed Al–Zr alloy and the HPT-processed CP Al in the temperature range of manifestation of the AIH and DIS effects are shown in Table 3.

## 4. Discussion

As is seen in Table 3, both in the Al–0.4Zr alloy and CP Al, annealing leads to a sharp decrease in the activation energy of the plastic flow in this temperature range. In HPT and HPT + AN + 0.25 HPT states, the *Q*-values are comparable, which points to recovery of the key microstructural features (most probably, the density of EGBDs) which are responsible for the implementation of plasticity in these states. Alloying by Zr leads to a decrease in the *Q*-value in the HPT and HPT + AN + 0.25 HPT from ~95 kJ/mol in CP Al to ~80 kJ/mol in the Al–0.4Zr alloy, which is associated with the presence of Zr atoms and, after annealing, nanoprecipitates in GBs in the HPT-processed alloy [20].

According to the model [11], the onset of plastic flow in HPT-processed Al occurs due to the emission of lattice dislocations (LDs) from GB triple junctions. In non-equilibrium GBs, GBD pile-ups form at triple junctions under an applied stress. The stress field from such pile-ups is added to the applied stress that means that a lower value of the latter is required to emit LDs. During annealing, GB relaxation occurs, accompanied by a decrease in the density of GBDs. The obtained estimates of *Q* can reflect the role of thermal activation processes that ensure the formation of such GBD pile-ups under loading. Following the discussion in [13], the thermally activated glide of GBDs in HPT-processed CP Al is controlled by the energy *E* of kink formation and migration along the GBD, where *E* ≈ 0.4–0.5 eV with the 0.43 eV value giving the best fit to the experimental data. It is worth noting that the *Q*-value measured in our experiments, ~95 kJ/mol ≈ 0.984 eV/at., corresponds well to the energy of a double kink, 2*E* ≈ 0.8–1.0 eV. One can speculate that the presence of Zr atoms, which are of 160 pm in radius as compared to 143 pm for Al, slightly diminish the effective interatomic distance in GB planes which leads to a corresponding decrease in the effective magnitude of the Burgers vectors of GBDs. As a result, the Peierls barrier *E*_P_ for their glide along the GBs and, therefore, the 2*E*~2*E*_P_^1/2^ [28] value must be noticeably smaller in the HPT-processed Al–0.4Zr alloy than in the case of CP Al. This can explain the diminished value of *Q* (~80 kJ/mol ≈ 0.829 eV/at.) for the alloy.

In the annealed state (HPT + AN), there are no (or very few) EGBDs for the formation of dislocation pile-ups, so the much weaker temperature dependence σ_0.2_ (*T*) in this state is apparently determined by other processes. An energy of 7.5 kJ/mol (0.08 eV/at.) in the case of CP Al is comparable with the activation energies of kink formation for LDs (*E*~0.09 eV in pure Al [28]). For the HPT + AN state of the Al–0.4Zr alloy, we obtained significantly higher values of 23 and 28 kJ/mol (0.24 and 0.29 eV/at.) for *Q*. Two reasons could be considered for explaining this effect. The first one is quite evident: it concerns the role of Zr atoms as stoppers for gliding LDs. Indeed, the characteristic energy of interaction of an LD with an impurity atom can be estimated as [29] ~0.1*Gb*^3^, where *G* and *b* are the shear modulus and the Burgers vector magnitude, respectively, in CP Al. With *G* = 27 GPa and *b* = 0.286 nm, we obtain ~0.39 eV which is of the same order of magnitude as *Q* in this case. The second reason concerns the role of Al_3_Zr nanoprecipitates that form at GBs under annealing and can serve as sources for LDs. The energy barrier for the dislocation emission could be associated with *Q* as well. We have postponed the discussion of this possibility until a forthcoming publication [30].

The measured values of activation energy ~95 kJ/mol for HPT-processed CP Al and ~80 kJ/mol for HPT-processed Al–0.4Zr are comparable with the value of ∼ 84 kJ/mol anticipated for GB diffusion in pure Al [31]. However, the difference in the behavior of *σ*_0.2_ (*T*) in the range of 193–293 K for the HPT and HPT + AN states may not be explained by GB diffusion, because the energy of grain boundary diffusion would rather increase in the annealed state (relaxed state, accompanied by a decrease in the free volume in the GB) but not drop to such small values (Table 3). As is known, non-equilibrium GBs can have a higher diffusivity in comparison to the equilibrium ones [32].

The observed weakening of the AIH and DIS effects with decreasing the temperature of tensile test may be explained on the base of model [11] discussed above and the assumption of the thermally-activated glide of EGBDs forming pile-ups at triple junctions of GBs, which emit LDs into grains. With a decrease in *T_def_*, the mobility of EGBDs also decreases and the dislocations have no time to form sufficiently strong pile-ups, hence their role in emitting LDs diminishes. At a certain lower *T_def_*, such pile-ups of EGBDs cannot form during the sample loading to the yield point, hence, the AIH and DIS effects cease to exist.

The extension of the AIH effect towards lower temperatures in the HPT-processed Al–Zr alloy compared to HPT-processed CP Al is in good agreement with the decrease in the *Q*-value. In the case of a lower activation energy, EGBDs take less time to form pile-ups at triple junctions during the sample’s loading. Therefore, the attenuation of the AIH effect occurs at a lower *T_def_* in the Al–Zr alloy compared to the CP Al. The fine dislocation structure in GBs and grains interior in the HPT state and the influence of deformation-heat treatment on it could be important subjects of further studies in the future as well as being the background for developing theoretical models explaining the revealed effects.

## 5. Conclusions

In the present paper, the effect of deformation-induced softening (DIS) accompanied by a significant increase in ductility in the UFG Al–Zr alloy has been demonstrated for the first time. The influence of deformation temperature in the range of 77–300 K on the DIS effect and the previously reported effect of annealing-induced hardening (AIH) has been studied. It has been shown that both the AIH and DIS effects weaken with a decrease in the deformation temperature. Alloying with Zr expands the temperature range of the AIH effect manifestation to lower temperatures as compared to the similar effect in CP Al. For example, the AIH effect still occurs at 193 K (the yield stress increase is Δ*σ*_0.2_ ≈ 46 MPa) in the Al–0.4Zr alloy, while it is totally suppressed in CP Al at similar temperature. The DIS effect completely disappears in the HPT-processed Al–Zr alloy at 193 K.

The strain-rate sensitivity coefficient at room temperature has been estimated for the Al–0.4Zr alloy in the HPT and HPT + AN + 0.25 HPT states as *m* ≈ 0.045. The estimates have been completed for the activation energy *Q* of the plastic flow in the states after HPT processing, after annealing, and after annealing and additional deformation for both the HPT-processed Al–Zr alloy and HPT-processed CP Al in the temperature range of manifestation of the AIH and DIS effects. In both of the materials, annealing leads to a sharp decrease in the *Q*-value in this temperature range. Doping by Zr results in a decrease of *Q* in the HPT and HPT + AN + 0.25 HPT states from ~95 kJ/mol in CP Al to ~80 kJ/mol in the Al–0.4Zr alloy, which is associated with the presence of Zr atoms and, after annealing, Al_3_Zr nanoprecipitates in GBs in the UFG Al–0.4Zr alloy. It has been speculated that these values of *Q* could be associated with the energy of double kink formation on the lines of GBDs gliding in the GB planes. The difference in the values of *Q* for the Al–0.4Zr alloy and CP Al could then be caused by the diminishing of the effective interatomic distance within GB planes due to the presence of Zr atoms. For the annealed state (HPT + AN), much smaller values of *Q* have been revealed (7.5 and 23–28 kJ/mol in CP Al and Al–0.4Zr alloy, respectively) which can be explained by a lack of extrinsic GB dislocations for the formation of dislocation pile-ups in GBs and the activation of the glide of lattice dislocations stored near or emitted from GBs. The big difference in the *Q*-values for the HPT + AN states in CP Al and the Al–0.4Zr alloy is probably due to either the role of Zr atoms as stoppers for gliding lattice dislocations or the energy barrier for the dislocation emission from Al_3_Zr nanoprecipitates that form at GBs in the Al–0.4Zr alloy under annealing.

## Figures and Tables

**Table 1:** Microstructure characterization results for Al–0.4Zr alloy in different structural states; *d*_av_—mean grain size, *f*_HAGB_—fraction of HAGBs, *θ*_av_—average GBs misorientation angle, *a*—lattice parameter, *D*_XRD_—average size of coherent scattering domains, *<ε*^2^ *>* ^1/2^—elastic microdistortion level, and *L*_dis_—dislocation density.

State	EBSD Data [9,14]			XRD Data			
	*d*_av_ (nm)	*f*_HAGB_ (%)	*θ*_av_ (grad)	*a* (Å)	*D*_XRD_ (nm)	*<ε*^2^*>*^1/2^, ×10^−5^	*L*_dis_, ×10^12^ (m^−2^)
HPT	835 ± 13	82	33.8	4.0505 ± 0.0001	570 ± 15	2.5 ± 0.02	5.2
HPT + AN	880 ± 15	86	39.8	4.0512 ± 0.0001	815 ± 15	0.9 ± 0.02	1.4
HPT + AN + 0.25 HPT	-	-	-	4.0515 ± 0.0001	495 ± 10	2.2 ± 0.01	5.4
HPT + AN + 0.75 HPT	-	-	-	4.0509 ± 0.0001	490 ± 10	2.3 ± 0.01	5.7

**Table 2:** Mechanical properties of Al–0.4Zr alloy in different structural states after tensile tests at different temperatures. *σ*_0.2_—yield stress, *σ*_UTS_—the ultimate tensile strength, *δ*—elongation to failure, and *δ*_1_—uniform elongation.

State	*T*_def_ (K)	*σ*_0.2_ (MPa)	*σ*_UTS_ (MPa)	*δ* (%)	*δ*_1_ (%)
Initial State	293	122 ± 2	131 ± 2	26 ± 2	4 ± 1
HPT	77	314 ± 3	367 ± 1	14 ± 1	1 ± 0.2
	193	239 ± 1	295 ± 1	12 ± 0.5	1.4 ± 0.2
	223	217 ± 3	265 ± 2	13 ± 0.5	1.5 ± 0.1
	253	186 ± 3	239 ± 1	16 ± 1	2.0 ± 0.7
	273	161 ± 1	212 ± 4	21 ± 1	4.0 ± 0.3
	293	130 ± 1	202 ± 1	22 ± 1	8.3 ± 0.7
HPT + AN	77	319 ± 21	375 ± 7	14 ± 1	0.8 ± 0.2
	193	285 ± 8	311 ± 1	12 ± 1	0.7 ± 0.1
	223	264 ± 2	296 ± 2	12 ± 0.5	0.9 ± 0.1
	253	253 ± 6	283 ± 5	11 ± 0.5	1.1 ± 0.1
	273	241 ± 2	268 ± 1	12 ± 0.2	1.2 ± 0.1
	293	223 ± 2	252 ± 1	13 ± 0.5	1.5 ± 0.1
HPT + AN + 0.25 HPT	77	338 ± 5	386 ± 4	12 ± 1	1.1 ± 0.2
	193	252 ± 2	304 ± 2	13 ± 1	1.6 ± 0.1
	223	218 ± 4	279 ± 2	16 ± 1	1.9 ± 0.2
	253	193 ± 1	248 ± 2	18 ± 1	2.7 ± 0.1
	273	172 ± 1	230 ± 2	20 ± 1	4.6 ± 0.1
	293	131 ± 3	213 ± 2	24 ± 1	9.4 ± 0.1

**Table 3:** Strain-rate sensitivity coefficient (*m*) and the activation energy (*Q*).

Material	State	*m*	*Q*_|ε = 0.2%_ (kJ/mol)	*Q*_|ε = 1%_ (kJ/mol)
Al–0.4Zr	HPT	0.045	82 ± 15	80 ± 10
	HPT + AN		23 ± 4	28 ± 5
	HPT + AN + 0.25 HPT	0.045	80 ± 17	80 ± 10
CP Al *	HPT	0.03 [24]	95 ± 15	
	HPT + AN		7.5	
	HPT + AN + 0.25 HPT		95 ± 17	

* The energy estimates are made based on the results of ref. [13].

## Data Availability

The raw and processed data required to reproduce these results are available by reasonable request.
